# Editorial: The convergence of AI, LLMs, and industry 4.0: enhancing BCI, HMI, and neuroscience research

**DOI:** 10.3389/fncom.2026.1780276

**Published:** 2026-02-03

**Authors:** Umer Asgher

**Affiliations:** 1 Laboratory of Human Factors and Automation in Aviation, Department of Air Transport, Faculty of Transportation Sciences, Czech Technical University in Prague (CTU), Prague, Czechia; 2 National Center of Artificial Intelligence (NCAI), National University of Sciences and Technology (NUST), Islamabad, Pakistan; 3 School of Interdisciplinary Engineering and Sciences (SINES), National University of Sciences and Technology (NUST), Islamabad, Pakistan

**Keywords:** AI ethics and governance, AI safety, brain–computer interface, EEG, human–computer interaction, large language models, LSTM, neuroergonomcis

The Research Topic “The Convergence of AI, LLMs, and Industry 4.0: Enhancing BCI, HMI, and Neuroscience Research” is anchored in a clear systems-level proposition based on: progress in BCI, neuroergonomics, and human–machine interaction, integration across sensing, cognition, interaction, and deployment infrastructure. In this framing, “convergence” is not a generic term for interdisciplinary work; it denotes a concrete engineering and scientific shift toward closed-loop human AI systems that can (i) measure human cognitive and affective states, (ii) reason and communicate through natural language interfaces that actively shape user behavior and decisions, and (iii) operate inside real operational environments governed by cyber-physical constraints, safety requirements, and accountability obligations.

Operationally, the convergence has three interlocking pillars. First, it requires AI models that infer and adapt to human state, moving beyond static prediction toward neuroadaptive control: models must handle non-stationary signals, inter-subject variability, and context dependence, and must deliver uncertainty-aware outputs that can be used for safe adaptation. Second, it requires LLM-centric interaction layers that serve as the cognitive interface between humans and complex systems supporting explanation, decision support, and interactive reasoning but also introducing new variables into the socio-technical system (trust calibration, cognitive offloading, overreliance, and changes in attentional allocation). Third, it requires Industry 4.0-grade deployment substrates: integration into cyber-physical systems with latency and reliability constraints; continuous monitoring for drift and failure modes; and governance mechanisms that are implementable, auditable, and resilient.

The four published articles (Edwards; Jiang et al.; Li et al.; Ramezani et al.) instantiate this convergence agenda from four complementary entry points that, when viewed together, form a coherent research mosaic. Edwards provides a conceptual and architectural argument that safe AI, especially when embedded in interactive settings that requires explicit computational machinery for perspective taking and value-grounded behavior, shifting alignment from a *post-hoc* constraint to a designed system layer. Jiang et al. bring the convergence into measurable neuroergonomics by framing LLM interaction as a modulator of cognition and demonstrating that assistance may alter neurophysiological signatures and perceived workload, motivating evaluation regimes where cognitive cost and attention dynamics become first-class outcomes. Li et al. ground the convergence in an Industry 4.0 clinical cyber-physical ecosystem by showing how modern NLP can strengthen safety surveillance for robotic surgery, illustrating the practical reality that deployment demands scalable monitoring and triage pipelines, not only sophisticated models. Ramezani et al. contribute a rigorous computational neuroscience methodology for interrogating internal representations in language models, treating them as analyzable cognitive systems and providing tools that can eventually be aligned with brain data to evaluate mechanistic correspondences rather than relying on performance metaphors.

Taken together, these contributions provide a credible cross-section of what “convergence” currently looks like in the literature that is multi-disciplinary and technically plausible, with clear building blocks spanning alignment, neurocognitive measurement, safety analytics, and representational analysis as shown in Figure 1. At the same time, they also illuminate the critical gap that “matters most for deployment”: the field is still largely assembling components, while the decisive next phase requires integration science shared benchmarks and evaluation protocols that jointly assess decision quality, cognitive workload, trust, overreliance risks, robustness to drift, and governance compliance within closed-loop systems operating in realistic cyber-physical contexts.

**Figure 1:**
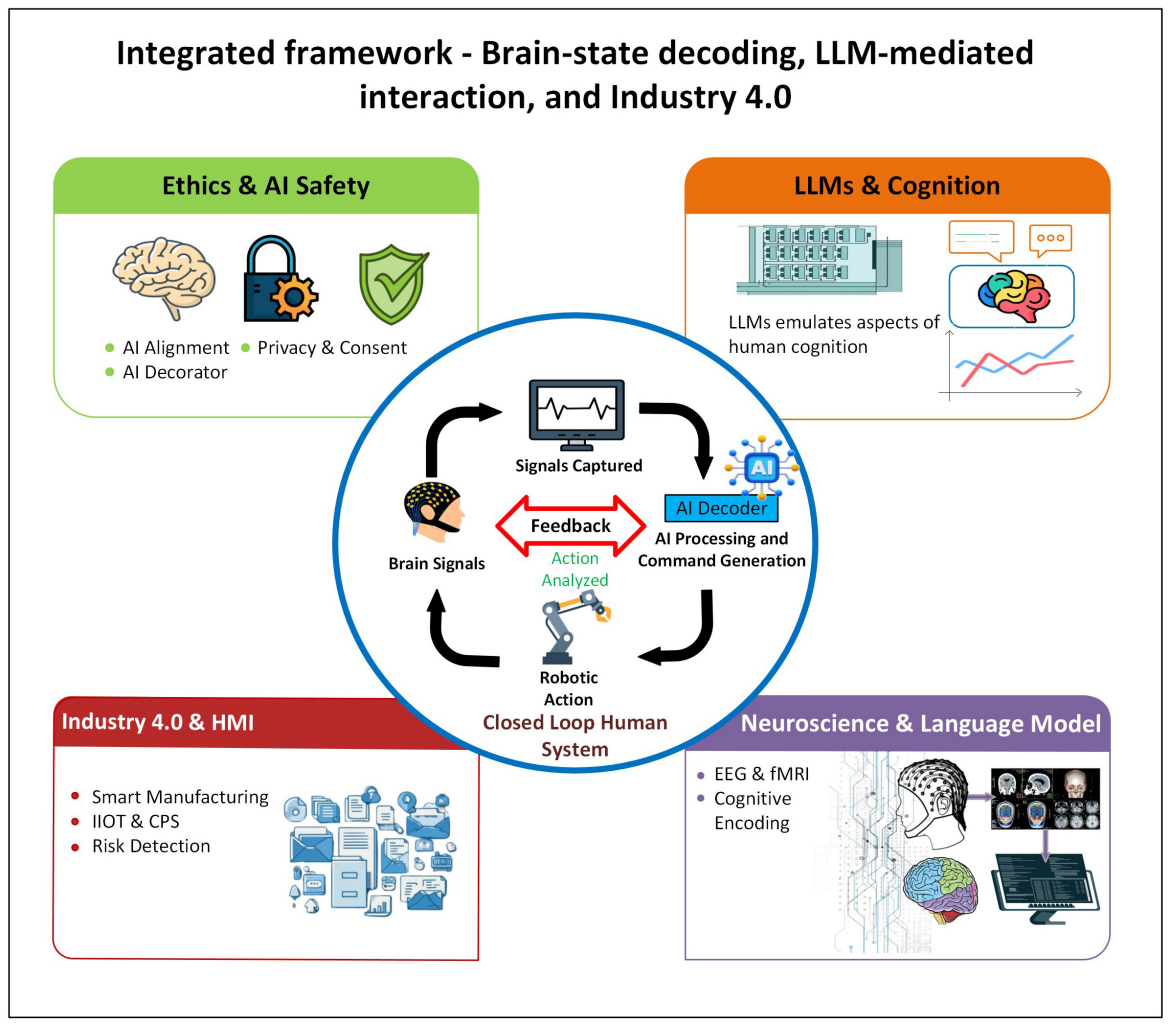
System-level convergence of Artificial Intelligence (AI), Large Language Models (LLMs), and Industry 4.0 for neuroadaptive human–machine systems.

## A unifying lens: convergence as a socio-technical control loop

A productive way to read the Topic and to situate the four contributions is to view AI/LLM-enabled HMI and neurotechnology as a closed-loop socio-technical system:

- **Interaction layer (LLM/HMI):** natural language and multimodal interfaces that influence cognition, attention, and decision behavior.
- **Measurement layer (BCI/neuroergonomics):** physiological and behavioral sensing that quantifies cognitive load, affect, and state transitions.
- **Inference and policy layer (AI/RL):** models that predict state, optimize assistance, and manage uncertainty.
- **Deployment layer (Industry 4.0/CPS):** integration into clinical/industrial workflows with safety monitoring, auditability, and governance.

The four articles map onto these layers in different proportions, and their value becomes clearer when interpreted as “components of a loop.”

## Contributions of articles within the convergence of Research Topic

- **Values, perspective-taking, and interpretability as alignment infrastructure (Edwards)**.

Edwards confronts a foundational concern that is often under-addressed in applied BCI/HMI work: what does it mean for an increasingly capable AI system to remain safe, context-sensitive, and socially compatible. The article proposes an observer-centric, functional-contextual, neuro-symbolic approach to alignment, explicitly targeting emergent Theory of Mind (ToM) as a computational capability rather than a philosophical label. The work is notable for insisting that alignment cannot be reduced to surface “safety prompts,” and instead requires explicit representational machinery: values specification (using ACT-inspired framing), utility estimation, and perspectival reasoning guiding agentic action (in the article's case, guiding LLM token selection).

From a convergence perspective, Edwards contributes two key ideas. First, it elevates perspective-taking and deictic relations as practical prerequisites for robust HMI, particularly in safety-critical environments where misinterpretation of intent is a primary failure mode. Second, it argues for structured interpretability via relational (hypergraph) representations of ToM-relevant relations, offering a conceptual pathway to auditing the “why” behind agent behavior. My view is that Edwards' most valuable contribution is not the breadth of its metaphors, but the insistence that alignment must be engineered as a visible layer of the system, not treated as an emergent byproduct of scale. In the Research Topic's context, this article anchors the “ethics and safety” pillar in an explicit computational vocabulary.

- **Neurocognitive impacts of LLM assistance measured in EEG (Jiang et al.)**

Jiang et al. move the convergence discussion into a measurable domain: how does LLM interaction modulate cognition during problem solving and decision-making. The article's framing is ambitious, positioning EEG as an objective lens on cognitive load and attentional engagement under LLM-assisted vs. solo reasoning. Methodologically, the article introduces an interaction-aware transformer framework (IALT) and an interaction-optimized reasoning strategy (IORS), with attention reweighting and reinforcement-style optimization intended to align model reasoning with interaction quality.

The article's empirical base is dominated by established affective EEG datasets (DEAP, AMIGOS, SEED, DREAMER) and conventional classification metrics, which risks construct-validity critiques if read too literally as “LLM cognition” evidence. However, the most on-topic and editorially important evidence is the article's pilot human study: across 12 participants performing problem-solving tasks with and without GPT-4 support, the LLM-assisted condition is associated with reduced frontal theta power (lower workload), increased P300 amplitude (enhanced attentional/decision integration), and lower NASA-TLX workload ratings. This is exactly the type of measurable neuroergonomics signal the Research Topic called for: not merely that LLMs can answer questions, but that they can shift the cognitive economics of reasoning for better or worse, under defined interaction conditions.

In my opinion, Jiang et al. are directionally correct: the next frontier is not “LLM capability” in isolation, but LLM-in-the-loop cognitive dynamics. Where the article is strongest is in providing quantifiable hooks (theta, P300, subjective workload) that future Industry 4.0 deployments can operationalize as constraints or triggers (e.g., assistance adaptation, throttling, escalation). Limitations may include demonstrating a full closed-loop deployment scenario (adaptive assistance policies validated in realistic operational tasks), and in separating the contribution of capacity vs. architectural novelty in benchmark improvements. Nonetheless, it is the clearest “BCI + LLM interaction” bridge in the set.

- **Industry 4.0-style safety analytics in a clinical robotic ecosystem (Li et al.)**

Li et al. contribute a pragmatically deployable artifact: a text classification pipeline that triages adverse events for the da Vinci surgical robot using MAUDE reports (2013–2023). The work is not BCI in the narrow sense, but it is highly aligned with the Research Topic's Industry 4.0 logic: cyber-physical systems (robotic surgery) generate complex, safety-critical event streams, and AI must reduce human burden while improving risk detection. The proposed Bert-BiLSTM-Att_dropout model reaches an average F1 of approximately 90.15 in the authors' benchmark set, outperforming simpler RNN baselines and a BERT-only approach.

The deeper significance of this article is not the novelty of the architecture *per se* (BERT + BiLSTM + attention + dropout is incremental in modern NLP), but the operational framing: harm vs. no-harm triage as a first-step surveillance action. In post-market monitoring, the value of such models depends on calibrated risk, temporal generalization, and explainability that supports regulatory workflows. In my opinion and the agenda, it sets is that future work must move beyond random splits into time-aware validation and cost-sensitive evaluation (false negatives for “harm” are not symmetric with false positives). Still, in a convergence portfolio, Li et al. supply the concrete “Industry 4.0 safety monitoring” dimension that purely cognitive or representational articles often lack.

- **Computational neuroscience of language models via construction-level representations (Ramezani et al.)**

Ramezani et al. sit at the most “computational neuroscience of AI” end of the spectrum. The article asks whether a recurrent language model (LSTM), trained only on next-word prediction, develops internal representations that separate argument structure constructions (transitive, ditransitive, caused-motion, resultative). A distinctive methodological choice is the use of controlled GPT-4 generated stimuli (2,000 sentences, balanced across constructions), coupled with a disciplined representational analysis stack: MDS and t-SNE visualizations plus GDV (Generalized Discrimination Value) computed in the original activation space. The key finding is that constructional separability is strongest in the final hidden layer (with slight reduction at the output layer) and is consistent with a broader principle: latent structure often becomes maximally linearly separable in penultimate representations.

For this Research Topic, the article does two important things. First, it legitimizes the “LLM era” as not only an engineering revolution but a scientific opportunity: models can be treated as objects of cognitive analysis, with internal geometry that can be interrogated. Second, it demonstrates a credible workflow for synthetic-stimulus control (with explicit caveats): synthetic data can enable targeted probing of representational hypotheses, provided researchers remain vigilant about template leakage and generalization beyond prompts. My view is that this article strengthens the Topic's intellectual foundation by providing a rigorous representational methodology that could, in future, be aligned with brain data (RSA/encoding models), thereby closing the loop between AI representations and neurophysiology.

- **Comparative synthesis:** The Research Topic cover (as mentioned in Figure 1), Safety, and alignment as system requirements, not afterthoughts: Edwards provides a conceptual architecture for value and perspective-aware AI; Li et al. operationalize safety monitoring in a real clinical robotic ecosystem. LLM interaction as a neurocognitive variable: Jiang et al. provide direct EEG evidence (theta, P300, workload ratings) that LLM assistance modulates cognitive effort and attention, supporting the Topic's for studying psychological and cognitive impacts. A methodological bridge between AI and neuroscience: Ramezani et al. show how language models can be analyzed with computational neuroscience tools, emphasizing structure in hidden states rather than only task performance.

## Conclusion

The four articles in this Research Topic show that “convergence” is a substantive research direction rather than a slogan, and that it is already taking shape through complementary contributions. Edwards argues that safe AI must be engineered with explicit mechanisms for perspective-taking, context sensitivity, and value-grounded utility, treating alignment as a designed system property. Jiang et al. provide neuroergonomic evidence that LLM assistance is cognitively consequential, demonstrating measurable shifts in workload and attentional signatures in EEG, which motivates evaluation beyond task accuracy toward cognitive cost and attentional dynamics during human and LLM collaboration. Li et al. anchor the convergence narrative in an Industry 4.0 clinical cyber-physical setting by showing how modern NLP can strengthen robotic-surgery safety surveillance via harm triage of adverse-event reports. Ramezani et al. contribute a rigorous computational neuroscience toolkit for probing internal representations in language models, positioning them as analyzable cognitive systems rather than purely black-box predictors.

Collectively, due to contribution of the Research Topic, field is assembling the parts of a neuroadaptive, Industry 4.0-ready human-AI-loop interaction, neurophysiological measurement, operational safety analytics, and alignment framing and the decisive advances that come from integration of these technologies.

